# Utility of ctDNA in predicting relapse in solid tumors after curative therapy: a meta-analysis

**DOI:** 10.1093/jncics/pkad040

**Published:** 2023-05-27

**Authors:** Abhenil Mittal, Consolacion Molto Valiente, Faris Tamimi, Massimo Di Iorio, Laith Al-Showbaki, David W Cescon, Eitan Amir

**Affiliations:** Division of Medical Oncology and Hematology, Department of Medicine, Princess Margaret Cancer Center, University of Toronto, ON, Canada; Division of Medical Oncology and Hematology, Department of Medicine, Princess Margaret Cancer Center, University of Toronto, ON, Canada; Division of Medical Oncology and Hematology, Department of Medicine, Princess Margaret Cancer Center, University of Toronto, ON, Canada; Division of Medical Oncology and Hematology, Department of Medicine, Princess Margaret Cancer Center, University of Toronto, ON, Canada; Division of Hematology and Medical Oncology, Department of Medicine, Jordan University Hospital and School of Medicine, The University of Jordan, Amman, Jordan; Division of Medical Oncology and Hematology, Department of Medicine, Princess Margaret Cancer Center, University of Toronto, ON, Canada; Division of Medical Oncology and Hematology, Department of Medicine, Princess Margaret Cancer Center, University of Toronto, ON, Canada

## Abstract

**Background:**

Presence of circulating tumor DNA (ctDNA) is prognostic in solid tumors treated with curative intent. Studies have evaluated ctDNA at specific “landmark” or multiple “surveillance” time points. However, variable results have led to uncertainty about its clinical validity.

**Methods:**

A PubMed search identified relevant studies evaluating ctDNA monitoring in solid tumors after curative intent therapy. Odds ratios for recurrence at both landmark and surveillance time points for each study were calculated and pooled in a meta-analysis using the Peto method. Pooled sensitivity and specificity weighted by individual study inverse variance were estimated and meta-regression using linear regression weighted by inverse variance was performed to explore associations between patient and tumor characteristics and the odds ratio for disease recurrence.

**Results:**

Of 39 studies identified, 30 (1924 patients) and 24 studies (1516 patients) reported on landmark and surveillance time points, respectively. The pooled odds ratio for recurrence at landmark was 15.47 (95% confidence interval = 11.84 to 20.22) and at surveillance was 31.0 (95% confidence interval = 23.9 to 40.2). The pooled sensitivity for ctDNA at landmark and surveillance analyses was 58.3% and 82.2%, respectively. The corresponding specificities were 92% and 94.1%, respectively. Prognostic accuracy was lower with tumor agnostic panels and higher with longer time to landmark analysis, number of surveillance draws, and smoking history. Adjuvant chemotherapy negatively affected landmark specificity.

**Conclusions:**

Although prognostic accuracy of ctDNA is high, it has low sensitivity, borderline high specificity, and therefore modest discriminatory accuracy, especially for landmark analyses. Adequately designed clinical trials with appropriate testing strategies and assay parameters are required to demonstrate clinical utility.

Adjuvant systemic therapy (chemotherapy; radiation; and, more recently, immunotherapy) improves outcomes for solid tumors; however, absolute incremental benefits of various therapies in many tumor types are modest (1-4). A large proportion of patients may be cured with surgical removal of the primary tumor alone. For most solid tumors, the decisions for adjuvant therapy are based on conventional clinical factors associated with risk of recurrence (1,5,6). In recent years, pathological response to neoadjuvant therapy and gene expression profiling assays (eg, Oncotype DX and MammaPrint in breast cancer) have been incorporated in standard adjuvant treatment decision making (7-10). However, these tools are based on population-based estimates of risk and remain imperfect, such that treatment decisions continue to rely on clinical risk factors and shared decision making between patients and clinicians (11,12).

To detect relapse after curative therapy, oncologists have relied on clinical, imaging, and blood-based tumor biomarkers; however, these are limited by their sensitivity and specificity and their ability to detect a recurrence before the development of incurable metastatic disease (13,14). Furthermore, studies that have tried to initiate systemic therapy preemptively based on tumor markers have failed to observe improved overall survival (15). Therefore, improved biomarkers are needed to identify patients who are at greater risk of recurrence in whom early intervention may improve outcomes.

For several hematologic malignancies, detection of minimal or measurable residual disease (MRD) in blood or bone marrow using flow cytometry, digital droplet polymerase chain reaction, or next-generation sequencing (NGS) has been established as a poor prognostic factor following induction therapy (16). Modification of therapy based on MRD detection is standard of care in acute lymphoblastic leukemia, acute promyelocytic leukemia, and chronic myelogenous leukemia and is under active investigation in acute myeloid leukemia and multiple myeloma. Given such actionability of MRD in hematological malignancies, biomarkers that could identify patients with solid tumors who harbor MRD might have utility in personalizing adjuvant therapy or preemptively treating patients in an effort to prevent or “intercept” the development of metastatic disease (17-19).

Circulating tumor DNA (ctDNA) is being extensively evaluated as a potential MRD marker in solid tumors. Since the first report of ctDNA detection in solid tumors in 1994 (20), major technological advancements have been made in the field, enabling a variety of new methods with improved analytical sensitivity and specificity. Several observational studies have identified the adverse prognostic impact of ctDNA detection following completion of curative intent therapy. These have variously employed single time point “landmark” analyses or strategies for repeated “surveillance” testing at defined intervals. Although these reports have contributed to an increasing understanding of MRD detection in the context of solid tumors, multiple questions remain unanswered. Importantly, test performance of ctDNA platforms across various solid tumors (as measured by sensitivity and specificity) is uncertain. Additionally, the magnitude of prognostic information is not well established.

The primary objective of our study was to quantify the prognostic value of ctDNA and determine the pooled sensitivity and specificity of ctDNA at both “landmark” and “surveillance” time points to inform the ongoing investigation of these different strategies. Secondarily, we aimed to explore sources of heterogeneity across different solid tumors and performed subgroup analyses to identify specific clinical characteristics or tumor types where ctDNA monitoring for relapse prediction may have differential evidence of clinical validity.

## Methods

### Literature review and study identification

The review and meta-analysis was conducted in accordance with Meta-Analysis of Observational studies in Epidemiology guidelines (21) (see Supplementary Table 1, available online). Two independent reviewers (A.M. and C.M.) searched MEDLINE (host: PubMed) to identify studies published between January 1, 2000, and May 7, 2022, and that evaluated the test performance and/or prognostic value of ctDNA measurement in solid tumors after curative intent therapy at landmark and/or surveillance time points. The following MeSH terms were used for the search: “circulating tumor DNA” and “cancer.” We restricted our search to studies performed in adults and reported in English. When multiple reports of 1 study were published, the most recent version with the longest follow-up was included in the analysis. We only included studies that reported the absolute number of patients with and without recurrence and their ctDNA status at the time of or before detection of recurrence. Risk of bias assessment was performed using the Newcastle Ottawa Scale (NOS) for observational studies (Supplementary Table 2, available online) (22). This validated scale assesses studies on the basis of selection, comparability, and outcomes of included studies and ranks studies as good, fair, or poor quality based on these parameters.

### Data extraction

Data were independently collected by 2 reviewers (A.M. and C.M.). Discrepancies were resolved by a third reviewer (E.A.). All data were extracted from primary publications and their associated online appendices. Collected data for all studies included summary study characteristics such as number of patients (N), tumor type, methodology for ctDNA testing (type of tests including NGS or polymerase chain reaction, number of genes and variants tracked, and limit of detection [when available]), type of panel used (tumor informed [if it used mutational information from a patient’s primary tumor to create a personalized assay] vs tumor agnostic [if a general panel was used without consideration of patient-specific tumor mutations]), median age, proportion of male sex, proportion of patients with advanced-stage disease at presentation, proportion of patients receiving neoadjuvant or adjuvant chemotherapy, proportion of patients with smoking history, median follow-up time, and lead time (time interval between ctDNA detection and radiological and/or clinical recurrence). We also extracted the number of patients with and without radiological or symptomatic recurrence and their corresponding ctDNA status as reported in the studies. For studies reporting on landmark analysis, time to landmark blood draw and timing of blood draw with respect to adjuvant chemotherapy were also noted. When reported, we also extracted the number of surveillance draws for studies reporting on surveillance time points. Sensitivity of ctDNA was defined as the percentage of patients who were ctDNA positive (either at landmark time point for landmark analyses or at any time point during surveillance analyses) before or at the time of recurrence among those who eventually had recurrent disease. Specificity was defined as percentage of patients who remained ctDNA negative and free of recurrence until last follow-up on the study.

### Data synthesis and statistical analysis

Sensitivity and specificity of ctDNA at both landmark and surveillance time points were calculated for each individual study. We calculated the variance of each study and used inverse variance to weigh the pooled sensitivity and specificity of ctDNA at both landmark and surveillance time points. We then calculated odds ratios (ORs) for disease recurrence at both landmark and surveillance time points for each study. Because recurrences were generally rare events, these were pooled in a meta-analysis using the Peto method (fixed effects) (23) because this method has been identified as the least biased and most powerful (24). Magnitude of heterogeneity was assessed using the I^2^ test. We performed multiple subgroup analyses for both landmark and surveillance time points to define the effect of primary tumor site, type of panel (tumor informed vs tumor agnostic), and timing of landmark sample with respect to adjuvant chemotherapy (pre- vs postchemotherapy). Differences between subgroups were evaluated using Mann-Whitney *U* test for 2 groups or Kruskal-Wallis test for more than 2 groups. Meta-regression using linear regression weighted by inverse variance was performed to explore associations between the natural logarithm for the odds ratio for disease recurrence and study level patient and tumor characteristics (median age, proportion of males, proportion with smoking history, proportion with advanced stage, proportion of patients receiving adjuvant or neoadjuvant chemotherapy, time to landmark blood draw [for landmark analysis], and number of surveillance blood draws [for surveillance analysis]). For meta-regression, statistical power was expected to be very low because the unit of measurement was individual studies of which fewer than 50 were expected to be included in the analyzable cohort. Therefore, we decided to make inference based on quantitative significance rather than statistical significance. The threshold for quantitative significance was defined using methods described by Burnand et al. (25), with a coefficient ß ≥ 0.28 considered quantitatively significant irrespective of statistical significance. However, tests for statistical significance were reported nominally. Meta-regression was also performed for sensitivity and specificity at both landmark and surveillance points. Sensitivity analyses were performed excluding studies with poor methodological quality for both landmark and surveillance analysis. All analyzes were performed using SPSS version 28.0 (IBM Corp, Armonk, NY, USA) and Review Manager v5.4. For meta-analysis, statistical significance was defined as *P* less than .05.

## Results

### Study characteristics

**Figure 1.**
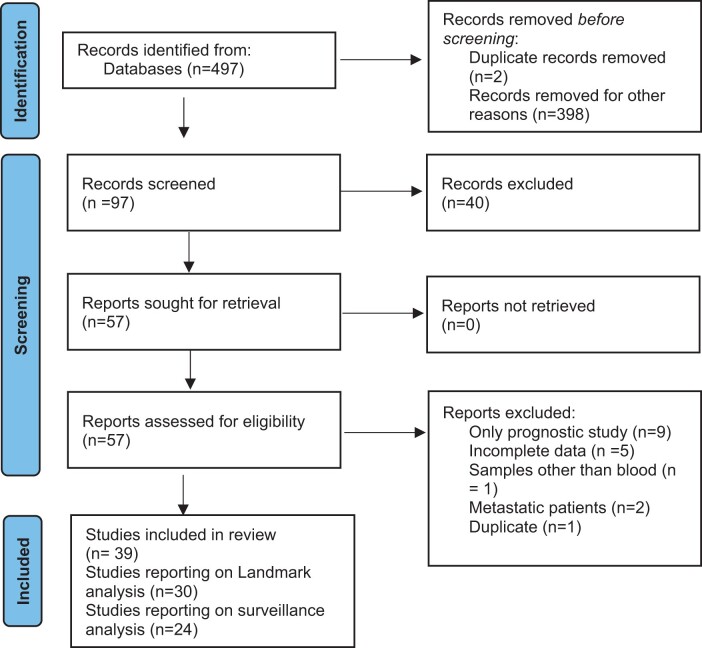
Flow diagram for study selection.

A total of 39 observational studies reporting on 2774 patients met the inclusion criteria (Figure 1). Of these studies, 30 studies (N = 1924) reported on landmark analyses and 24 studies (N = 1516) reported on surveillance analyses; 15 studies (N = 1161) reported on both landmark and surveillance time points. The characteristics of the included studies are detailed in Table 1 and Supplementary Tables 3 and 4 (available online). Among both landmark and surveillance analysis, much of the data were derived from studies in colorectal cancer (14 studies [N = 1284] for landmark and 9 studies, N = 769 for surveillance). Most studies used a tumor-informed approach to ctDNA surveillance (25 of 30 studies [83.3%] of landmark studies and 21 of 24 studies [87.5%] of surveillance studies). Further details of ctDNA testing strategies are provided in Supplementary Table 5 (available online). Four studies did not provide data on use of adjuvant chemotherapy (26-29), and 1 study each reported on consolidation (30) and adjuvant immunotherapy (31); hence, they were excluded from the subgroup analysis of adjuvant chemotherapy. Mean baseline ctDNA positivity (defined as any measurable ctDNA) was 69.8% of patients in landmark studies and 72.8% of patients in surveillance studies. Overall, 12 studies each reporting on landmark and surveillance analysis were of good methodological quality as determined by NOS.

**Table 1.** Summary of included studies^a^

Study details	Primary site	Study design	Panel type	No of patients	Baseline ctDNA positivity (%)	Landmark sensitivity	Landmark specificity	Surveillance sensitivity	Surveillance specificity
Azad et al., 2020 (69)	Gastric/esophagus	P	Tumor agnostic	45	60	77.7%	95.45%	NA	NA
Yang et al., 2020 (70)	Gastric/esophagus	P	Tumor agnostic	46	50	41.2%	100%	84.2%	96%
Benhaim et al., 2021 (71)	Colorectal	P	Tumor agnostic	184	27.5	27.6%	92.9%	NA	NA
Chen et al., 2021 (72)	Colorectal	P	Tumor agnostic	240	64.2	60%	96.3%	82.6%	94.1%
Diehl et al., 2008 (27)	Colorectal	P	Tumor agnostic	18	100	100%	80%	NA	NA
Henriksen et al., 2022 (73)	Colorectal	P	Tumor informed	160	91	42.1%	96.1%	88%	98.8%
Jin et al., 2020 (50)	Colorectal	P	Tumor agnostic	82	89	55%	71.7%	NA	NA
Khakoo et al., 2019 (74)	Colorectal	P	Tumor agnostic	23	74	75%	100%	NA	NA
McDuff et al., 2021 (75)	Colorectal	P	Tumor informed	29	34.6	66.6%	100%	NA	NA
Parikh et al., 2021 (76)	Colorectal	P	Tumor agnostic	84	88	55.6%	95.3%	70.4%	95.7%
Reinert et al., 2019 (51)	Colorectal	P	Tumor informed	125	88.5	70%	88.1%	87.5%	98.3%
Scholer et al., 2017 (77)	Colorectal	P	Tumor agnostic	26	74	60%	100%	100%	100%
Tarazona et al., 2019 (53)	Colorectal	P	Tumor agnostic	94	63.8	44.4%	89.65%	77.7%	96.5%
Tie et al., 2019a (78)	Colorectal	P	Tumor informed	96	NA	42%	94.4%	NA	NA
Tie et al., 2019b (79)	Colorectal	P	Tumor informed	159	77	47.8%	94.11%	NA	NA
Wang et al., 2021 (54)	Colorectal	P	Tumor agnostic	91	88.7	57.4%	80%	NA	NA
Fakih et al., 2022 (80)	Colorectal	R	Tumor agnostic	48	100	NA	NA	53.3%	100%
Tie et al., 2016 (81)	Colorectal	P	Tumor informed	178	No	NA	NA	40.7%	98%
Wang et al., 2019 (82)	Colorectal	NGS	Tumor informed	58	No	NA	NA	100%	93.8%
Chaudhuri et al., 2017 (83)	Lung	R	Tumor agnostic	40	93	94%	100%	100%	100%
Chen et al., 2019 (49)	Lung	P	Tumor agnostic	27	83.9	85.7%	70%	NA	NA
Moding et al., 2020 (30)	Lung	R	Tumor agnostic	28	75	75%	93%	100%	100%
Waldeck et al., 2022 (57)	Lung	P	Tumor informed	21	57	50%	100%	55.5%	83.3%
Zviran et al., 2020 (29)	Lung	P	Tumor informed	22	NA	100%	70.6%	NA	NA
Abbosh et al., 2017 (55)	Lung	P	Tumor informed	24	48	NA	NA	92.9%	90%
Peng et al., 2020 (32)	Lung	P	Tumor agnostic	77	No	NA	NA	63.3%	61.9%
Qiu et al., 2021 (84)	Lung	P	Tumor informed	89	No	NA	NA	79.4%	92.7%
Coombes et al., 2019 (85)	Breast	P	Tumor informed	49	100	55.6%	100%	88.9%	100%
Garcia Murillas, 2019 (85)	Breast	P	Tumor informed	101	51	NA	NA	79%	91.7%
Garcia Murillas et al., 2015 (86)	Breast	P	Tumor informed	55	78	50%	96%	80%	96.4%
Parsons et al., 2020	Breast	R	Tumor informed	142	Yes	NA	NA		
Olsson et al., 2015 (87)	Breast	P	Tumor agnostic	20	No	NA	NA	92.8%	100%
Groot et al., 2019 (56)	Pancreas	P	Tumor informed	59	49	37%	92.8%	90%	88%
Jiang et al., 2020 (28)	Pancreas	P	Tumor informed	27	66.7	57.1%	96.3%	NA	NA
Lee et al., 2019 (88)	Pancreas	P	Tumor agnostic	42	62	56.5%	100%	NA	NA
Sausen et al., 2015 (52)	Pancreas	P	Tumor agnostic	24	43	57.1%	75%	NA	NA
Tan et al., 2019 (31)	Melanoma	P	Tumor informed	68	36	44.8%	100%	66.6%	92.1%
Christensen et al., 2019 (89)	Bladder	P	Tumor informed	66	NA	45.8%	97.1%	NA	NA
Flach et al., 2022 (26)	Head and neck	P	Tumor informed	17	93	NA	NA	100%	100%

a NA = not available; NGS = next-generation sequencing; P = prospective; PCR = polymerase chain reaction; R = retrospective.

### Landmark analysis

**Figure 2.**
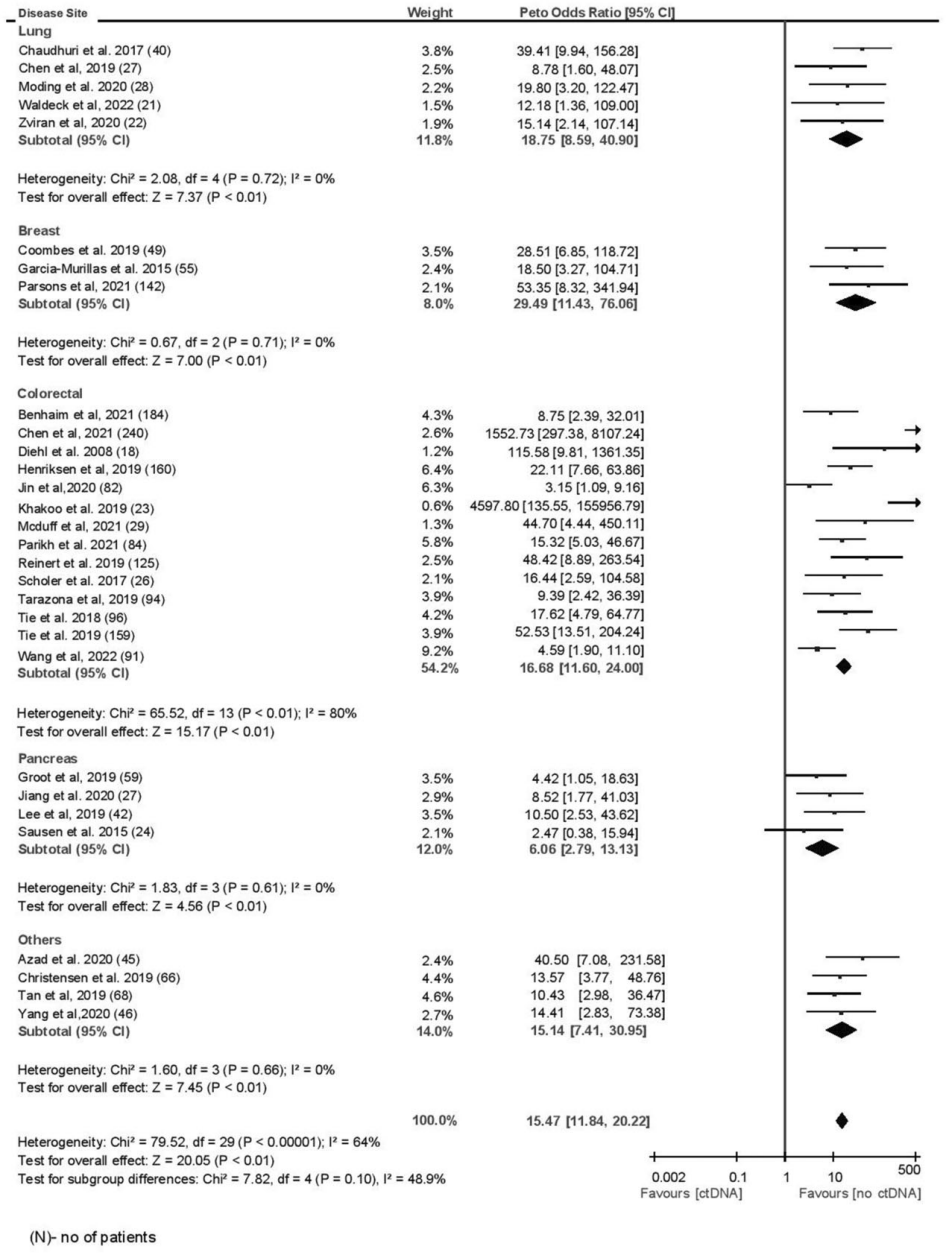
Landmark time analysis by disease site.

**Figure 3.**
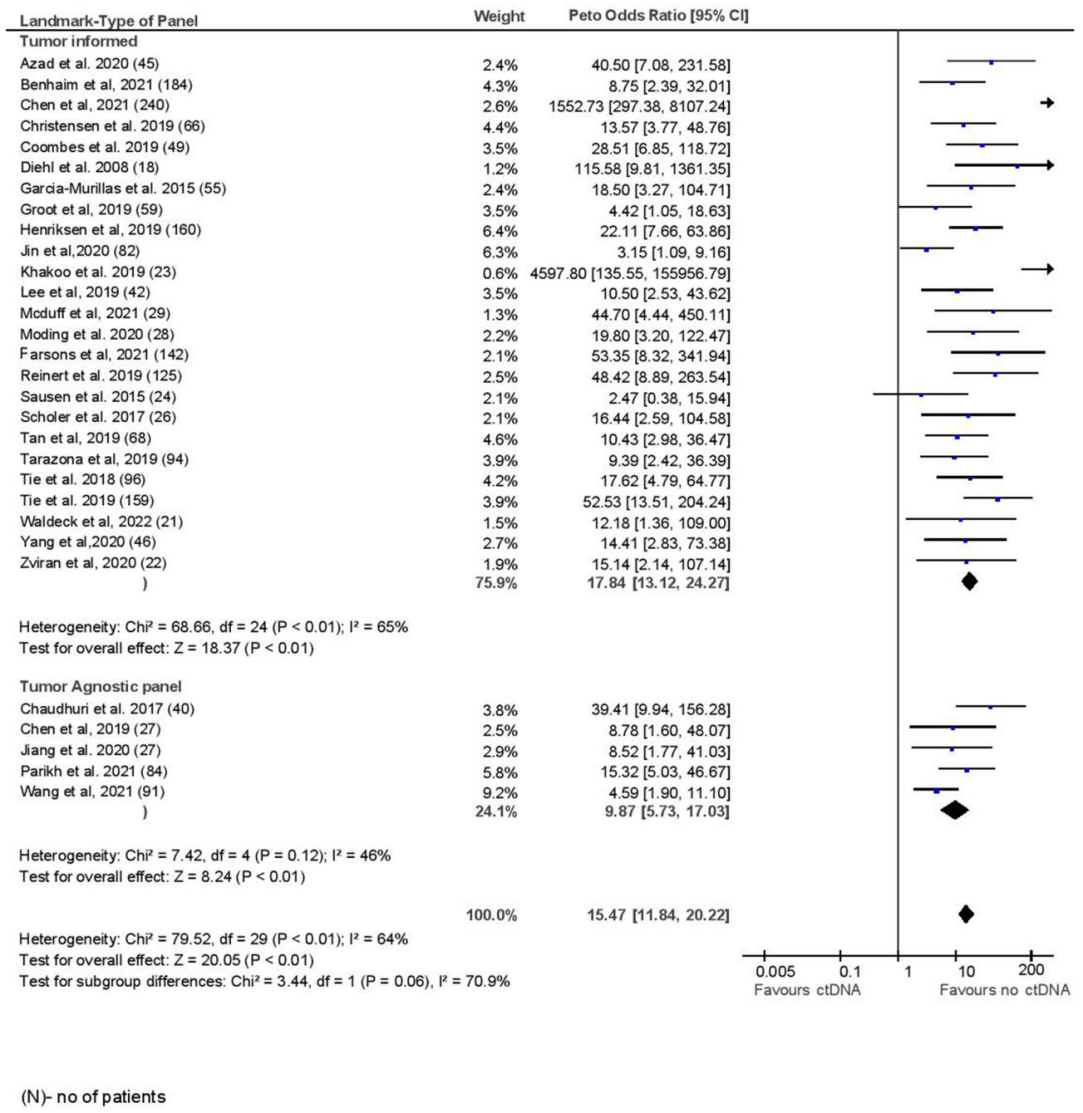
Landmark time analysis by type of panel.

The mean sensitivity of ctDNA at landmark time point was 58.3% with specificity of 92.0%. The sensitivity was lower in a sensitivity analysis considering studies of only good methodological quality, whereas specificity was similar (sensitivity 47.9% and specificity 95.1%). The median time to landmark testing after completion of all definitive therapy was 28 days (range = 3-426 days). At a median follow-up of 24.7 months (range = 12.5-84 months), among those with a positive ctDNA test and a clinical or radiographic recurrence, the lead time was 5.1 months (range = 2-11.5 months). The odds ratio for recurrence for a positive ctDNA test at landmark time point was 15.47 (95% confidence interval [CI] = 11.84 to 20.22, I^2^ = 64%). The odds ratio remained similar when only studies of good methodological quality were considered (OR = 22.64, 95% CI = 15.26 to 33.59, *P* < .001, I^2^ = 72%). There was no statistically significant difference in prognostic accuracy by primary tumor site (*P* = .10) (Figure 2). There was higher prognostic accuracy for tumor-informed panels compared with tumor agnostic panels, which approached but did not meet statistical significance (OR = 17.84 [95% CI = 13.12 to 24.27] vs 9.87 [95% CI = 5.73 to 17.03, *P* = .06]; Figure 3). Landmark samples taken after adjuvant chemotherapy showed higher prognostic accuracy compared with samples analyzed before adjuvant chemotherapy, which again approached but did not meet statistical significance (OR = 24.48 [95% CI = 10.61 to 56.46] vs 10.94 [95% CI = 7.90 to 16.39], *P* = .09; Supplementary Figure 1, available online). Subgroup analysis for pooled sensitivity and specificity did not show any significant differences (Supplementary Table 6, available online). In meta-regression analysis, higher stage (β = 0.38, *P* = .07) and longer time to landmark (β = 0.45, *P* = .02) were associated with quantitatively better prognostic accuracy, whereas prognostic accuracy was quantitatively lower in older patients (β = −0.41, *P* = .03) and those receiving neoadjuvant chemotherapy (β = −0.51, *P* = .24; see Table 2). Meta-regression results were similar if only colorectal cancer studies were considered; however, time to landmark analysis lost quantitative significance (β = 0.17, *P* = .58) (Supplementary Table 7, available online). Meta regression results for sensitivity and specificity are shown in Supplementary Table 8 (available online). Visual inspection of funnel plot did not reveal any publication bias (Supplementary Figure 2, available online).

**Table 2.** Meta regression for odds ratio at landmark and surveillance

	Landmark			Surveillance		
Variable	Beta coefficient	*P*	No. of studies	Beta coefficient	*P*	No. of studies
Median age	−0.41	.035	26	0.065	.697	21
Proportion of males	−0.23	.24	27	−0.276	.225	24
Smoking	0.25	.68	5	0.549	.259	6
≥Stage III	0.38	.07	23	0.201	.438	17
Time to landmark	0.45	.02	26	−	−	−
No. of surveillance draws	−	−	−	0.362	.184	15
% Receiving adjuvant chemotherapy	−0.18	.4	21	0.411	.090	15
% Receiving neoadjuvant chemotherapy	−0.51	.24	7	0.188	.722	6
Baseline ctDNA positivity	0.14	.48	26	0.241	.293	21

### Surveillance analysis

**Figure 4.**
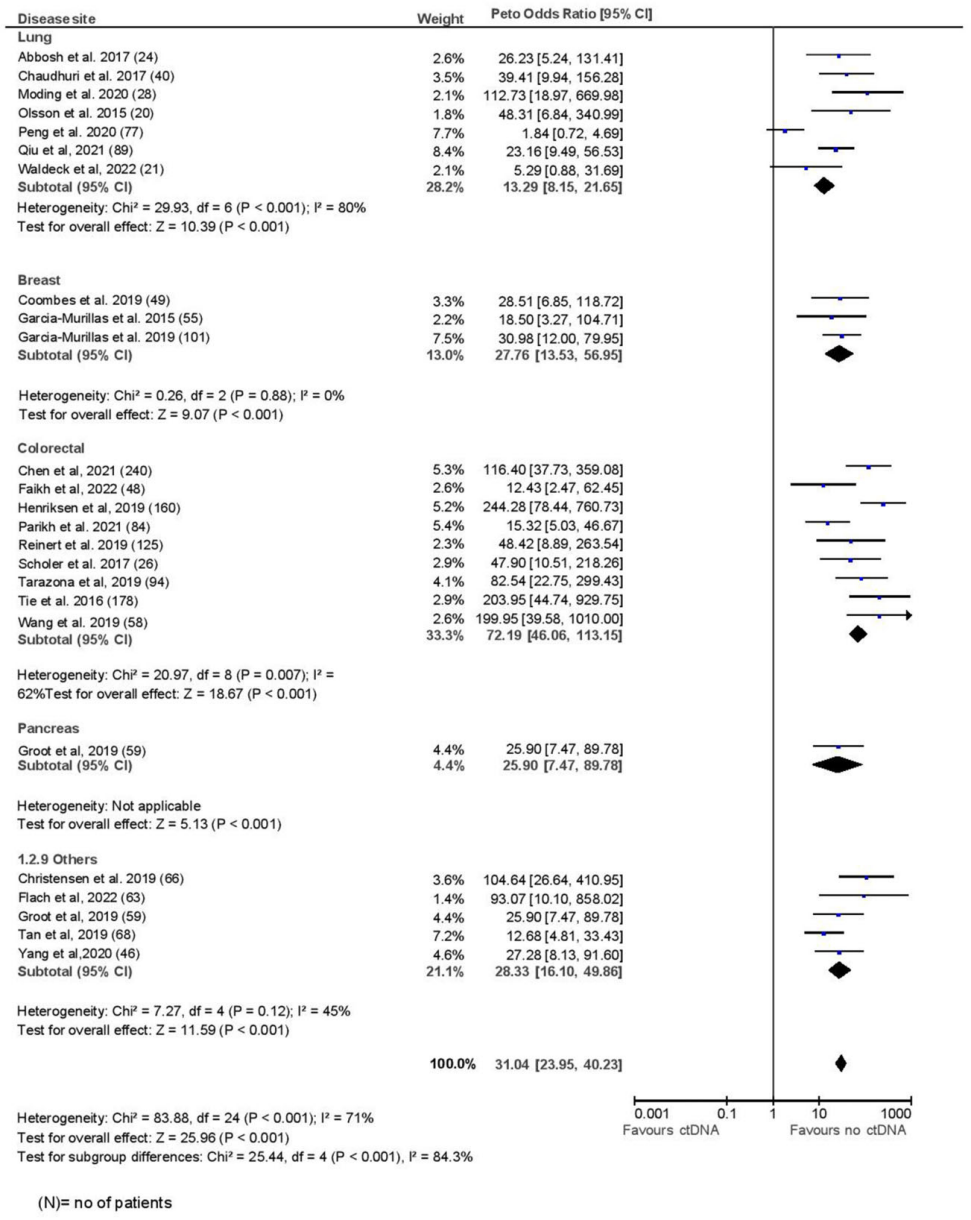
Surveillance analysis by disease site.

**Figure 5.**
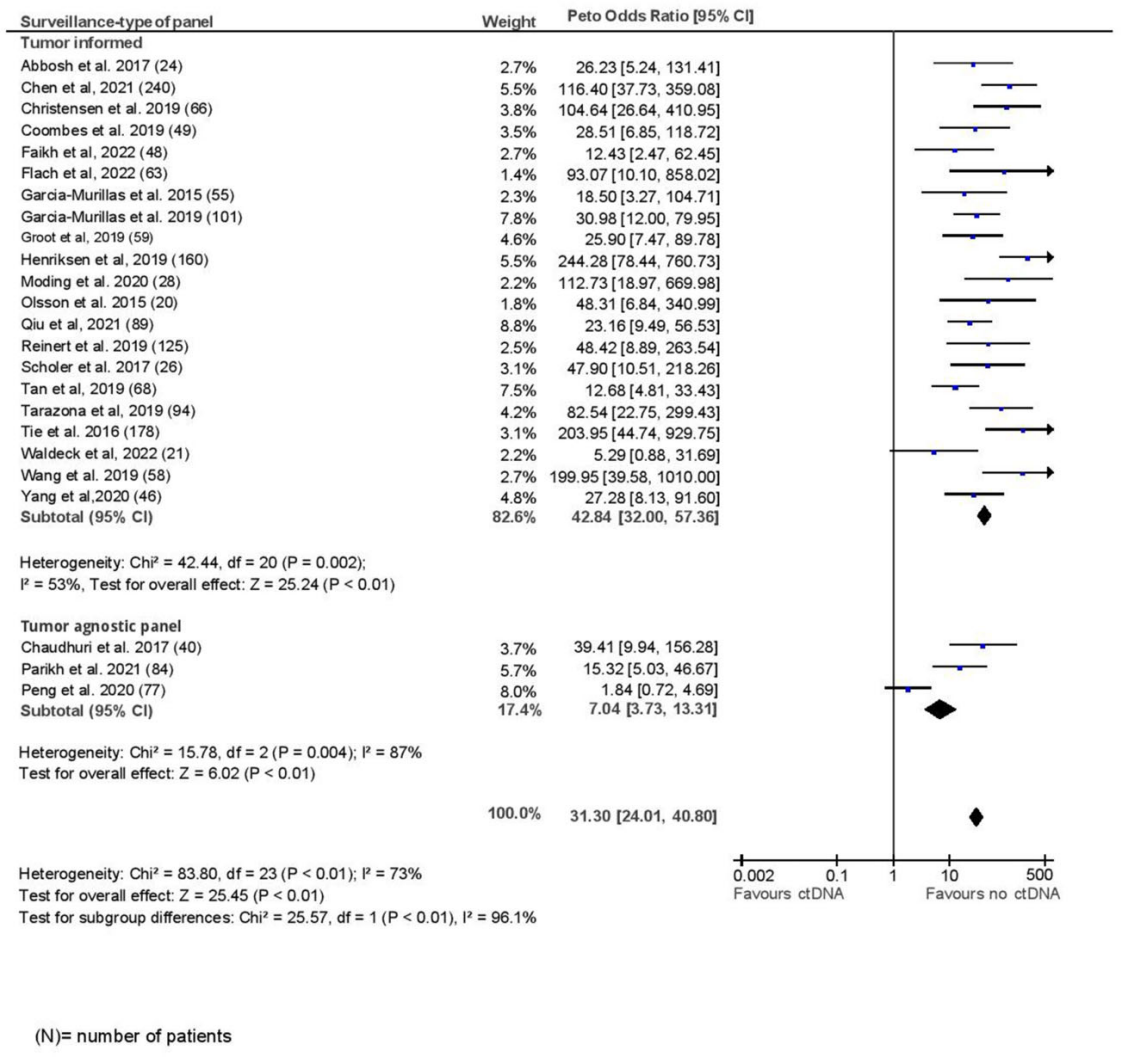
Surveillance analysis by type of panel.

The mean pooled sensitivity for surveillance analysis was 82.2%, with a corresponding specificity of 94.1%. These estimates were slightly lower in a sensitivity analysis of studies with good methodological quality on NOS (sensitivity, 77.4%; specificity, 92.6%). The median lead time between ctDNA positivity and clinical or radiologic recurrence among those with recurrence was 5.5 months (range = 0.7-12.6 months, median follow-up = 29.1 months). The median number of surveillance draws was 8 (range = 5-12). The odds for recurrence were significantly higher if ctDNA was positive during any surveillance time point (OR = 31.04, 95% CI = 23.95 to 40.23, I^2^ = 71%). Excluding studies of poor quality did not change the odds ratio substantially (OR = 32.29, 95% CI = 22.85 to 45.64, *P* < .001). Prognostic accuracy was lower for lung cancer and better for colorectal cancer compared with breast or other tumor sites (lung: OR = 13.29, 95% CI = 8.15 to 21.65; breast: OR = 27.76, 95% CI = 13.53 to 56.95; colorectal: OR = 72.19, 95% CI = 46.06 to 113.15; other: OR = 28.33, 95% CI = 16.10 to 49.86, *P*_difference_ < .001; Figure 4). Excluding the study by Peng et al. (32), which had the worst prognostic accuracy among lung cancer studies, the within-group differences remained significant, with highest accuracy for colorectal cancer (*P* = .03; Supplementary Figure 3, available online). Tumor-informed panels were associated with better prognostic accuracy than tumor agnostic panels, as observed in a landmark analysis (OR = 42.84, 95% CI = 32 to 57.36 vs 7.04, 95% CI = 3.73 to 13.31, *P* ≤ .001; Figure 5). Meta-regression showed quantitatively better prognostic accuracy with smoking (β = 0.55, *P* = .26), greater number of surveillance draws (β = 0.36, *P* = .18), and in patients receiving adjuvant chemotherapy (β = 0.41, *P* = .09; Table 2). No significant differences were observed in subgroup analysis for sensitivity and specificity (Supplementary Table 6, available online). Meta-regression for pooled sensitivity and specificity at surveillance time points is shown in Supplementary Table 9 (available online). No publication bias was observed by visual inspection of the funnel plot (Supplementary Figure 4, available online).

## Discussion

The value of ctDNA as a biomarker for tumor burden, treatment response, and prognosis has been established in patients with advanced solid tumors (33). In an attempt to integrate this promising biomarker into clinical decision making for solid tumors treated with curative intent, various observational studies have been performed to evaluate test performance and clinical validity for the detection of MRD in predicting future relapse. Because these studies have been limited individually by small sample sizes, event rates, and heterogeneity, we performed a pooled analysis to synthesize the evidence for the use of ctDNA surveillance in patients with solid tumors treated with curative intent therapy. We observed that positive ctDNA is prognostic for recurrence after curative treatment when tested either once or serially. Although sensitivity for predicting relapse was generally low, especially when tested at a single time point, specificity was higher albeit at a level in which clinical utility may be marginal. Generally, specificity of cancer surveillance tests needs to be very high so as to not expose patients who are unlikely to have recurrence to the substantial toxicity of anticancer therapy. It could be argued that specificity in the lower 90% range is borderline. However, specificity data need to be interpreted in the context of limitations of testing methodology, use of adjuvant chemotherapy, and variable follow-up duration. Furthermore, with most studies performed in colorectal cancer, caution is suggested in generalization of these data to other tumor sites. Of note, these estimates did not significantly change in a sensitivity analysis based on methodological quality of individual studies. Of interest, prognostic accuracy was better for tumor informed compared with tumor agnostic approaches, especially for surveillance time point. Furthermore, chemotherapy delivered either in the adjuvant or neoadjuvant setting affected specificity of landmark testing. Finally, the timing of ctDNA analysis with respect to adjuvant chemotherapy appeared important when landmark testing was used.

ctDNA detection at any time point after curative therapy was a strong predictor for relapse across solid tumor sites with a magnitude of effect greater than has been demonstrated for individual clinical and pathological prognostic factors (34-36). In this study, we also provide pooled estimates for magnitude of prognostic effect that can be used to counsel patients enrolled in clinical trials about optimizing adjuvant therapy based on ctDNA (37-39). On the basis of data reported in this analysis, randomization of ctDNA-positive patients who do not have symptoms or imaging evidence of recurrence to placebo in the presence of such high odds of recurrence presents a considerable challenge and may present a hurdle for recruitment to these trials. Alternative strategies including treatment escalation in ctDNA-positive patients and deescalation in those who are negative are being evaluated (40-42). However, given the low sensitivity (58.3%) in the landmark setting, which was further compromised with neoadjuvant chemotherapy, such approaches appear generally insufficient to support treatment deescalation in scenarios where a well-established role for adjuvant therapy exists (1,43,44).

Adjuvant chemotherapy affected specificity of ctDNA testing at a landmark time point. This is expected given the efficacy of adjuvant chemotherapy in eliminating residual disease in a proportion of patients. This has important implications for trials evaluating treatment escalation based on ctDNA positivity (40,45), which may result in overtreatment of a fraction of patients who otherwise would have been cured with standard of care alone. This also questions the optimal timing of ctDNA testing after curative intent surgery. We observed that the odds ratio for detecting relapse was better in patients who had “delayed landmark testing” (after adjuvant chemotherapy) with better prognostic accuracy as more time from curative surgery elapsed and all upfront standard therapy had been completed. Similar findings were observed for serial testing, where both sensitivity and specificity were improved with greater number of surveillance draws and in patients who had received chemotherapy in the adjuvant setting. It is plausible that patients having detectable ctDNA after adjuvant chemotherapy (at 1 or multiple time points) represent a population at highest risk of relapse and may be most suited for treatment escalation in the adjuvant setting. This group may also be most appropriate for trials of preemptive treatment for metastatic disease before detection of overt relapse (39,46).

Considering both landmark and serial surveillance testing, between 6% and 8% of patients may have a false-positive ctDNA result. Previous studies have shown that detection of variants related to clonal hematopoiesis could lead to false-positive results, especially at lower variant allele fractions typically detected in MRD setting (47,48). In our meta-analysis, for studies having limited specificity at landmark and surveillance time points (<90%) (27,29,32,49-57), only 5 definitely excluded clonal hematopoiesis of indeterminate potential (CHIP) by paired analysis of peripheral mononuclear cells. Moreover, among the landmark studies, median follow-up times for studies not exploring CHIP were shorter than the median for the entire cohort. This could have further affected specificity and led to false-positive results. In most of these studies (5 of 9 landmark studies), adjuvant chemotherapy was delivered after landmark testing, which could have further compromised specificity. Repeat testing to confirm a positive ctDNA result before clinical decision making could represent a potential strategy to improve specificity but may not account for false positives because of poor assay design, incomplete clinical follow-up, or elimination of residual disease with treatment.

To improve sensitivity and specificity of ctDNA detection, there has been a gradual shift toward using tumor-informed compared with tumor-agnostic approaches (58,59). In our analysis, we observed that prognostic accuracy of ctDNA was significantly better in studies that used a tumor-informed approach, an observation consistent in both landmark and surveillance analyses. The higher sensitivity and specificity of the tumor-informed approach and in turn higher prognostic accuracy are likely driven by the ability to track a larger number of specific cancer-derived variants in the plasma and better ability to exclude variants related to CHIP (55). Although such tumor-informed panels are promising, potential limitations include the requirement for tumor tissue for bespoke panel design and longer turnaround times, which may be particularly important when adjuvant therapy decisions need to be made. Interestingly, in the recently published DYNAMIC phase 2 randomized trial of ctDNA-guided adjuvant therapy in stage II colon cancer where a tumor-informed approach was used (61), ctDNA results were available to clinicians only 8-10 weeks after surgery. Previous studies have demonstrated that delaying initiation of adjuvant chemotherapy beyond 6-8 weeks in colorectal cancer can result in worse survival (62,63). Therefore, efforts to minimize turnaround time when a tumor-informed approach is pursued should be prioritized, and using presurgical biopsy material to design assays is a potential solution (64). This could also eliminate the noise introduced by neoadjuvant chemotherapy–induced clonal variations and may help design more homogenous assays. Immediate postsurgical specimen collection could also be an alternative because time to landmark testing did not seem to influence prognostic accuracy for colorectal cancer in our analysis. Newer approaches incorporating DNA methylation or whole-genome sequencing are also being developed for detection and measurement of ctDNA and may complement the assessment of MRD (60,65).

We observed that pooled estimates for prognostic accuracy were lower in studies evaluating ctDNA in lung cancer and higher for colorectal cancer compared with other tumor sites, especially when tested serially. This could have been driven by lower sensitivity and specificity seen in the study by Peng et al. (32), which used a tumor-agnostic approach and included a higher proportion of early-stage lung cancer patients. Excluding data from this study resulted in better prognostic accuracy for patients with lung cancer. The more aggressive biology and higher recurrence risk seen with lung cancer compared with colon cancer could account for these differences; however, heterogeneity in testing strategies and lower sensitivity of testing platforms in different studies limit definitive tumor-specific conclusions. More prospective data from larger cohort studies will be required to elucidate whether any true site-specific differences exist.

A higher prognostic accuracy was observed for patients with smoking, especially for surveillance testing. Of 6 studies that reported data on smoking status in the surveillance setting, 5 were performed in patients with lung cancer. Pivotal studies have shown that smoking is associated with a 10-fold higher mutational load compared with nonsmokers in lung cancer (66,67), which might account for higher probability of detection by NGS panels. This could also be driven by the association between smoking and adverse oncogenic mutations (eg, K-RAS) and subsequent higher risk of recurrence (49). However, given the small number of studies reporting on smoking status, the power of the analysis is limited to draw any definitive conclusions.

Our study has some limitations. All the studies included in the meta-analysis were observational studies with heterogenous study designs, ctDNA testing platforms, and varying definitions for ctDNA positivity both presurgery and for detection of MRD after curative therapy. This could potentially affect pooled analyses. Additionally, most of the data were derived from studies performed in colorectal cancer, with few studies and small number of patients in other tumor types (melanoma, bladder, gastroesophageal cancer, head and neck), thus limiting any disease site–specific conclusions.

In summary, sensitivity of ctDNA as a biomarker for MRD appears to be low, especially if measured at a single landmark time point. Although newer methods may partially address this issue, the phenomenon persists even in most recent studies that were published after our search was completed (68). In the studies included in our analysis, the tumor-informed approach improved prognostic accuracy but may be limited by issues of tissue availability and turn-around time. Whether emerging tumor agnostic approaches can improve sensitivity remains to be determined. Although sensitivity is improved with repeated surveillance measurements, it is unlikely to influence adjuvant chemotherapy decisions, which are currently made in a short time frame after locoregional therapy. However, ctDNA surveillance may enable future strategies for delayed systemic therapy. The impact of preemptive therapy based on ctDNA detected relapse in absence of radiological or clinical relapse on long-term cancer outcomes needs to be demonstrated in clinical trials before it can become standard of care. Additionally, specific methods and approaches for any interventional studies need to be carefully described, and the oncology community needs to be educated that different ctDNA assays cannot be used interchangeably.

## Supplementary Material

pkad040_Supplementary_Data

## Data Availability

The data extracted from studies can be made available on request from corresponding author.
