# Supplementary material for: Utility of ctDNA in predicting relapse in solid tumors after curative therapy: a meta-analysis
Source: JNCI Cancer Spectr. 2023 May 27;7(4):pkad040. doi: 10.1093/jncics/pkad040 (PMC10317487; doi:10.1093/jncics/pkad040)

References:

1. Azad TD, Chaudhuri AA, Fang P, Qiao Y, Esfahani MS, Chabon JJ, et al. Circulating Tumor DNA Analysis for Detection of Minimal Residual Disease After Chemoradiotherapy for Localized Esophageal Cancer. Gastroenterology. 2020 Feb;158(3):494-505.e6.

2. Benhaim L, Bouché O, Normand C, Didelot A, Mulot C, Le Corre D, et al. Circulating tumor DNA is a prognostic marker of tumor recurrence in stage II and III colorectal cancer: multicentric, prospective cohort study (ALGECOLS). Eur J Cancer. 2021 Dec;159:24–33.

3. Chaudhuri AA, Chabon JJ, Lovejoy AF, Newman AM, Stehr H, Azad TD, et al. Early Detection of Molecular Residual Disease in Localized Lung Cancer by Circulating Tumor DNA Profiling. Cancer Discov. 2017 Dec;7(12):1394–403.

4. Chen K, Zhao H, Shi Y, Yang F, Wang LT, Kang G, et al. Perioperative Dynamic Changes in Circulating Tumor DNA in Patients with Lung Cancer (DYNAMIC). Clin Cancer Res. 2019 Dec 1;25(23):7058–67.

5. Chen G, Peng J, Xiao Q, Wu HX, Wu X, Wang F, et al. Postoperative circulating tumor DNA as markers of recurrence risk in stages II to III colorectal cancer. Journal of Hematology & Oncology. 2021 May 17;14(1):80.

6. Coombes RC, Page K, Salari R, Hastings RK, Armstrong A, Ahmed S, et al. Personalized Detection of Circulating Tumor DNA Antedates Breast Cancer Metastatic Recurrence. Clin Cancer Res. 2019 Jul 15;25(14):4255–63.

7. Diehl F, Schmidt K, Choti MA, Romans K, Goodman S, Li M, et al. Circulating mutant DNA to assess tumor dynamics. Nat Med. 2008 Sep;14(9):985–90.

8. Garcia-Murillas I, Schiavon G, Weigelt B, Ng C, Hrebien S, Cutts RJ, et al. Mutation tracking in circulating tumor DNA predicts relapse in early breast cancer. Sci Transl Med. 2015 Aug 26;7(302):302ra133.

9. Groot VP, Mosier S, Javed AA, Teinor JA, Gemenetzis G, Ding D, et al. Circulating Tumor DNA as a Clinical Test in Resected Pancreatic Cancer. Clin Cancer Res. 2019 Aug 15;25(16):4973–84.

10. Henriksen TV, Tarazona N, Frydendahl A, Reinert T, Gimeno-Valiente F, Carbonell-Asins JA, et al. Circulating Tumor DNA in Stage III Colorectal Cancer, beyond Minimal Residual Disease Detection, toward Assessment of Adjuvant Therapy Efficacy and Clinical Behavior of Recurrences. Clin Cancer Res. 2022 Feb 1;28(3):507–17.

11. Jiang J, Ye S, Xu Y, Chang L, Hu X, Ru G, et al. Circulating Tumor DNA as a Potential Marker to Detect Minimal Residual Disease and Predict Recurrence in Pancreatic Cancer. Front Oncol. 2020 Jul 30;10:1220.

12. Jin S, Zhu D, Shao F, Chen S, Guo Y, Li K, et al. Efficient detection and post-surgical monitoring of colon cancer with a multi-marker DNA methylation liquid biopsy. Proc Natl Acad Sci U S A. 2021 Feb 2;118(5):e2017421118.

13. Khakoo S, Carter PD, Brown G, Valeri N, Picchia S, Bali MA, et al. MRI Tumor Regression Grade and Circulating Tumor DNA as Complementary Tools to Assess Response and Guide Therapy Adaptation in Rectal Cancer. Clin Cancer Res. 2020 Jan 1;26(1):183–92.

14. Lee B, Lipton L, Cohen J, Tie J, Javed AA, Li L, et al. Circulating tumor DNA as a potential marker of adjuvant chemotherapy benefit following surgery for localized pancreatic cancer. Ann Oncol. 2019 Sep 1;30(9):1472–8.

15. McDuff SGR, Hardiman KM, Ulintz PJ, Parikh AR, Zheng H, Kim DW, et al. Circulating Tumor DNA Predicts Pathologic and Clinical Outcomes Following Neoadjuvant Chemoradiation and Surgery for Patients With Locally Advanced Rectal Cancer. JCO Precision Oncology. 2021 Nov;(5):123–32.

16. Moding EJ, Liu Y, Nabet BY, Chabon JJ, Chaudhuri AA, Hui AB, et al. Circulating Tumor DNA Dynamics Predict Benefit from Consolidation Immunotherapy in Locally Advanced Non-Small Cell Lung Cancer. Nat Cancer. 2020 Feb;1(2):176–83.

17. Parikh AR, Van Seventer EE, Siravegna G, Hartwig AV, Jaimovich A, He Y, et al. Minimal Residual Disease Detection using a Plasma-only Circulating Tumor DNA Assay in Patients with Colorectal Cancer. Clin Cancer Res. 2021 Oct 15;27(20):5586–94.

18. Reinert T, Henriksen TV, Christensen E, Sharma S, Salari R, Sethi H, et al. Analysis of Plasma Cell-Free DNA by Ultradeep Sequencing in Patients With Stages I to III Colorectal Cancer. JAMA Oncol. 2019 Aug 1;5(8):1124–31.

19. Sausen M, Phallen J, Adleff V, Jones S, Leary RJ, Barrett MT, et al. Clinical implications of genomic alterations in the tumour and circulation of pancreatic cancer patients. Nat Commun. 2015 Jul 7;6:7686.

20. Schøler LV, Reinert T, Ørntoft MBW, Kassentoft CG, Árnadóttir SS, Vang S, et al. Clinical Implications of Monitoring Circulating Tumor DNA in Patients with Colorectal Cancer. Clinical Cancer Research. 2017 Sep 14;23(18):5437–45.

21. Tan L, Sandhu S, Lee RJ, Li J, Callahan J, Ftouni S, et al. Prediction and monitoring of relapse in stage III melanoma using circulating tumor DNA. Ann Oncol. 2019 May 1;30(5):804–14.

22. Tarazona N, Gimeno-Valiente F, Gambardella V, Zuñiga S, Rentero-Garrido P, Huerta M, et al. Targeted next-generation sequencing of circulating-tumor DNA for tracking minimal residual disease in localized colon cancer. Ann Oncol. 2019 Nov 1;30(11):1804–12.

23. Tie J, Cohen JD, Wang Y, Christie M, Simons K, Lee M, et al. Circulating Tumor DNA Analyses as Markers of Recurrence Risk and Benefit of Adjuvant Therapy for Stage III Colon Cancer. JAMA Oncol. 2019 Dec 1;5(12):1710–7.

24. Tie J, Cohen JD, Wang Y, Li L, Christie M, Simons K, et al. Serial circulating tumour DNA analysis during multimodality treatment of locally advanced rectal cancer: a prospective biomarker study. Gut. 2019 Apr;68(4):663–71.

25. Waldeck S, Mitschke J, Wiesemann S, Rassner M, Andrieux G, Deuter M, et al. Early assessment of circulating tumor DNA after curative-intent resection predicts tumor recurrence in early-stage and locally advanced non-small-cell lung cancer. Mol Oncol. 2022 Jan;16(2):527–37.

26. Wang DS, Yang H, Liu XY, Chen ZG, Wang Y, Fong WP, et al. Dynamic monitoring of circulating tumor DNA to predict prognosis and efficacy of adjuvant chemotherapy after resection of colorectal liver metastases. Theranostics. 2021;11(14):7018–28.

27. Yang J, Gong Y, Lam VK, Shi Y, Guan Y, Zhang Y, et al. Deep sequencing of circulating tumor DNA detects molecular residual disease and predicts recurrence in gastric cancer. Cell Death Dis. 2020 May 11;11(5):1–9.

28. Zviran A, Schulman RC, Shah M, Hill STK, Deochand S, Khamnei CC, et al. Genome-wide cell-free DNA mutational integration enables ultra-sensitive cancer monitoring. Nat Med. 2020 Jul;26(7):1114–24.

29. Christensen E, Birkenkamp-Demtröder K, Sethi H, Shchegrova S, Salari R, Nordentoft I, et al. Early Detection of Metastatic Relapse and Monitoring of Therapeutic Efficacy by Ultra-Deep Sequencing of Plasma Cell-Free DNA in Patients With Urothelial Bladder Carcinoma. JCO. 2019 Jun 20;37(18):1547–57.

30. Parsons HA, Rhoades J, Reed SC, Gydush G, Ram P, Exman P, et al. Sensitive Detection of Minimal Residual Disease in Patients Treated for Early-Stage Breast Cancer. Clin Cancer Res. 2020 Jun 1;26(11):2556–64.

31. Abbosh C, Birkbak NJ, Wilson GA, Jamal-Hanjani M, Constantin T, Salari R, et al. Phylogenetic ctDNA analysis depicts early-stage lung cancer evolution. Nature. 2017 Apr 26;545(7655):446–51.

32. Fakih M, Sandhu J, Wang C, Kim J, Chen YJ, Lai L, et al. Evaluation of Comparative Surveillance Strategies of Circulating Tumor DNA, Imaging, and Carcinoembryonic Antigen Levels in Patients With Resected Colorectal Cancer. JAMA Netw Open. 2022 Mar 1;5(3):e221093.

33. Flach S, Howarth K, Hackinger S, Pipinikas C, Ellis P, McLay K, et al. Liquid BIOpsy for MiNimal RESidual DiSease Detection in Head and Neck Squamous Cell Carcinoma (LIONESS)—a personalised circulating tumour DNA analysis in head and neck squamous cell carcinoma. Br J Cancer. 2022 May;126(8):1186–95.

34. Garcia-Murillas I, Chopra N, Comino-Méndez I, Beaney M, Tovey H, Cutts RJ, et al. Assessment of Molecular Relapse Detection in Early-Stage Breast Cancer. JAMA Oncol. 2019 Oct 1;5(10):1473–8.

35. Olsson E, Winter C, George A, Chen Y, Howlin J, Tang MHE, et al. Serial monitoring of circulating tumor DNA in patients with primary breast cancer for detection of occult metastatic disease. EMBO Mol Med. 2015 Aug;7(8):1034–47.

36. Peng M, Huang Q, Yin W, Tan S, Chen C, Liu W, et al. Circulating Tumor DNA as a Prognostic Biomarker in Localized Non-small Cell Lung Cancer. Front Oncol. 2020 Sep 15;10:561598.

37. Qiu B, Guo W, Zhang F, Lv F, Ji Y, Peng Y, et al. Dynamic recurrence risk and adjuvant chemotherapy benefit prediction by ctDNA in resected NSCLC. Nat Commun. 2021 Nov 19;12(1):6770.

38. Tie J, Wang Y, Tomasetti C, Li L, Springer S, Kinde I, et al. Circulating tumor DNA analysis detects minimal residual disease and predicts recurrence in patients with stage II colon cancer. Sci Transl Med. 2016 Jul 6;8(346):346ra92.

39. Wang Y, Li L, Cohen JD, Kinde I, Ptak J, Popoli M, et al. Prognostic Potential of Circulating Tumor DNA Measurement in Postoperative Surveillance of Nonmetastatic Colorectal Cancer. JAMA Oncol. 2019 Aug 1;5(8):1118–23.

Supplementary Table 1: MOOSE Checklist for Meta-analyses of Observational Studies

Item No	Recommendation	Reported on Page No
Reporting of background should include		
1	Problem definition	4, 5
2	Hypothesis statement	5
3	Description of study outcome(s)	5
4	Type of exposure or intervention used	5
5	Type of study designs used	5
6	Study population	5
Reporting of search strategy should include		
7	Qualifications of searchers (eg, librarians and investigators)	6
8	Search strategy, including time period included in the synthesis and key words	6
9	Effort to include all available studies, including contact with authors	6
10	Databases and registries searched	6
11	Search software used, name and version, including special features used (eg, explosion)	6
12	Use of hand searching (eg, reference lists of obtained articles)	Not applicable
13	List of citations located and those excluded, including justification	Table 1 provides list of included citations and figure 1 provides a flow diagram of reasons of study exclusions
14	Method of addressing articles published in languages other than English	Only included English language articles- page 6
15	Method of handling abstracts and unpublished studies	Only published literature included-6
16	Description of any contact with authors	None
Reporting of methods should include		
17	Description of relevance or appropriateness of studies assembled for assessing the hypothesis to be tested	6
18	Rationale for the selection and coding of data (eg, sound clinical principles or convenience)	6- based on hypothesis and included only those studies that had actual recurrence and ctDNA data available
19	Documentation of how data were classified and coded (eg, multiple raters, blinding and interrater reliability)	6
20	Assessment of confounding (eg, comparability of cases and controls in studies where appropriate)	6- risk of bias assessment by Newcastle Ottawa scale which assess adjustment for confounding
21	Assessment of study quality, including blinding of quality assessors, stratification or regression on possible predictors of study results	6- assessment of study quality of Newcastle Ottawa scale
22	Assessment of heterogeneity	Page 7
23	Description of statistical methods (eg, complete description of fixed or random effects models, justification of whether the chosen models account for predictors of study results, dose-response models, or cumulative meta-analysis) in sufficient detail to be replicated	Page 7
24	Provision of appropriate tables and graphics	Table 1- baseline characteristics<br>Table2- metaregression results<br>Figure 1- flow diagram of the study<br>Figure 2 and 3 represent statistically significant subgroup analysis

		Other result tables are presented in supplementary
Reporting of results should include		
25	Graphic summarizing individual study estimates and overall estimate	Individual study estimates are summarised in table 1. Overall study estiamtes are summarised in results- page 9 and 10
26	Table giving descriptive information for each study included	See Table 1
27	Results of sensitivity testing (eg, subgroup analysis)	Page 9 and 10- results section<br>Figure 2 and 3<br>Figure S2<br>Table S6
28	Indication of statistical uncertainty of findings	Heterogeneity estimates have been included, page 6 and 7

Item No	Recommendation	Reported on Page No
Reporting of discussion should include		
29	Quantitative assessment of bias (eg, publication bias)	Page 10,11 in results- we did a qualitative visual assessment as Egger's test is usually underpowered to detect statistical significance.
30	Justification for exclusion (eg, exclusion of non-English language citations)	We did not include this in main discussion as we did not think it was warranted. English language studies comprehensively cover literature and a non-english language search was not deemed necessary as most studies included in the review were fairly recent
31	Assessment of quality of included studies	A comment has been made in discussion and quality assessment has been included as a table in supplementary- page 11
Reporting of conclusions should include		
32	Consideration of alternative explanations for observed results	Discussed throughout discussion- page 11-15
33	Generalization of the conclusions (ie, appropriate for the data presented and within the domain of the literature review)	16
34	Guidelines for future research	16
35	Disclosure of funding source	24

Supplementary Table 2: Risk of Bias Assessment (Newcastle Ottawa Scale)

Studies	NOS- Selection	NOS- Comparability	NOS-outcomes	Overall Quality
Azad et al, 2020 (1)	3	1	1	poor
Benhaim et al, 2021 (2)	3	2	3	Good
Chaudhuri et al, 2017 (3)	3	1	2	Good
Chen et al, 2019 (4)	3	0	2	Poor
Chen et al, 2021(5)	3	1	2	Good
Coombes et al, 2019 (6)	3	0	2	Poor
Diehl et al, 2008 (7)	3	0	2	Poor
Garcia Murillas et al, 2015 (8)	3	2	1	Poor
Groot et al, 2019 (9)	3	2	2	Good
Henriksen et al, 2022 (10)	3	2	2	Good
Jiang et al, 2020 (11)	3	0	1	Poor
Jin et al, 2020 (12)	3	0	1	Poor
Khakoo et al, 2019(13)	3	0	2	Poor
Lee et al, 2019 (14)	3	0	2	Poor
McDuff et al, 2021 (15)	3	0	2	Poor
Moding et al, 2020 (16)	2	0	1	Poor
Parikh et al, 2021(17)	3	0	1	Poor
Reinert et al, 2019 (18)	3	1	2	Good
Sausen et al, 2015(19)	3	0	1	Poor
Scholer et al, 2017 (20)	2	0	1	Poor
Tan et al, 2019 (21)	3	2	3	Good
Tarazona et al, 2019 (22)	3	2	2	Good
Tie et al, 2019 (24)	3	2	2	Good
Tie et al, 2019 (23)	3	2	2	Good

Waldeck et al, 2022 (25)	2	0	1	Poor
Wang et al, 2021 (26)	3	2	1	Poor
Yang et al, 2020 (27)	3	0	2	Poor
Zviran et al, 2020 (28)	2	0	1	Poor
Christensen et al, 2019 (29)	3	2	2	Good
Abbosh et al, 2017 (31)	3	0	1	Poor
Fakih et al, 2022 (32)	2	1	2	Fair
Flach et al, 2022 (33)	2	0	1	Poor
Garcia Murillas, 2019 (34)	3	1	2	Good
Olsson et al, 2015 (35)	2	0	1	Poor
Peng et al, 2020 (36)	3	1	2	Good
Qiu et al, 2021 (37)	3	2	1	Poor
Tie et al, 2016 (38)	3	2	2	Good
Wang et al, 2019 (39)	3	0	2	Poor
Parsons et al, 2021 (30)	3	2	2	Good

Supplementary Table 3: Landmark Studies

Study details	Primary site	Testing methodology	Panel type	No of patients	Median Age (Years)	Smoking (%)	Proportion of males (%)	Proportion of >StageIII (%)	Primary treatment	Proportion receiving adjuvant chemo (%)	Median Time to landmark analysis (days)	Landmark sample pre or post adjuvant	Sensitivity (%)	Specificity (%)	Median lead time (months)
Azad et al, 2020(1)	Gastric esophagus	NGS	Tumor informed	45	67	64.4	88.9	71	Radiation	0	86	NA	77.7	95.4	3.8
Yang et al, 2020 (27)	Gastroesophageal	NGS	Tumor informed	46	54	NA	83	50	surgery	98	28	pre	41.17	100	6
Chen et al, 2021(5)	Colorectal	NGS	Tumor informed	240	60	NA	56.7	53.3	surgery	72.5	5	pre	60	96.3	5
Diehl et al, 2008 (7)	Colorectal	PCR	Tumor informed	18	59.8	NA	44	94	surgery	NA	34	NA	100	80	NA
Henriksen et al, 2022 (10)	Colorectal	NGS	Tumor informed	160	NA	NA	59.3	NA	surgery	86.9	14	Pre	42.1	96.1	10
Jin et al, 2020 (12)	Colorectal	PCR	Tumor informed	82	66	NA	64.6	50	surgery	85.3	14	pre	55	71.7	NA
Khakoo et al, 2019 (13)	Colorectal	PCR	Tumor informed	23	59	NA	61.7	87.2	surgery	0	47	NA	75	100	2.6
Benhaim et al, 2021 (2)	Colorectal	PCR	Tumor informed	184	66.7	NA	57.8	45.5	surgery	52.4	5	pre	27.6	92.9	3.1
Wang et al, 2021 (26)	Colorectal	NGS	Tumor agnostic	91	59	NA	51.6	NA	surgery	92	38	pre	57.44	80	NA
McDuff et al,	Colorectal	NGS	Tumor	29	54	NA	52	79	surgery	38	90	NA	66.6	100	NA

2021 (15)			informed												
Parikh et al, 2021(17)	Colorectal	NGS	Tumor agnostic	84	60	NA	60.7	66.6	surgery	45	28	post	55.6	95.3	NA
Reinert et al, 2019 (18)	Colorectal	NGS	Tumor informed	125	70	NA	58.4	64.8	surgery	77	30	pre	70	88.1	8.7
Tarazona et al, 2019 (22)	Colorectal	PCR	Tumor informed	94	71	NA	64.9	41	surgery	43.3	NA	pre	44.4	89.6	11.5
Tie et al, 2019 (23)	Colorectal	NGS	Tumor informed	96	64	NA	51	100	surgery	100	42	pre	42	94.4	NA
Scholer et al, 2017 (20)	Colorectal	PCR	Tumor informed	26	NA	NA	NA	NA	surgery	32.6	30	pre	60	100	9.4
Tie et al, 2019 (24)	Colorectal	NGS	Tumor informed	159	62	NA	67.3	78	surgery	64.1	49	post	47.8	94.1	NA
Chaudhuri et al, 2017 (3)	Lung	NGS	Tumor Agnostic	40	66.5	88	68	64	Radiation	70	120	NA	94	100	5.2
Chen et al, 2019 (4)	Lung	NGS	Tumor Agnostic	27	65	57.7	69		surgery	53.8	3	pre	85.7	70	NA
Waldeck et al, 2022 (25)	Lung	NGS	Tumor informed	21	70	100	67	86.7	surgery	42.8	14	pre	50	100	2.5
Zviran et al, 2020 (28)	Lung	NGS	Tumor informed	22	66	NA	46.1	NA	surgery	NA	17	NA	100	70.58	NA
Moding et al, 2020 (16)	Lung	NGS	Tumor informed	28	68.5	80	53.8	90.8	Radiation	0	7	NA	75	93	4.1
Coombes et al, 2019 (6)	Breast	NGS	Tumor Informed	49	57	NA	0	67	surgery	65	120	post	55.6	100	8.9
Parsons et al, 2021 (30)	Breast	NGS	Tumor Informed	142	35	NA	0	27	Surgery	91.6	426	Post	19	100	NA

Garcia Murillas et al, 2015 (8)	Breast	PCR	Tumor Informed	55	NA	NA	0	NA	surgery	0	21	NA	50	96	7.9
Groot et al, 2019 (9)	Pancreas	PCR	Tumor Informed	59	70	NA	53	27	surgery	41	24	pre	37	92.8	2.8
Jiang et al, 2020 (11)	Pancreas	NGS	Tumor Informed	27	62	NA	63	18.5	surgery	NA	7	pre	57.1	92.3	NA
Lee et al, 2019 (14)	Pancreas	PCR	Tumor informed	42	66	NA	62.1	NA	surgery	78.3	45	1	56.5	100	NA
Sausen et al, 2015(19)	Pancreas	PCR	Tumor informed	24	69	NA	62.4	0	surgery	86.1	NA	NA	57.1	75	6.5
Tan et al, 2019 (21)	Melanoma	PCR	Tumor informed	68	57	NA	66	100	surgery	24	14	pre	44.8	100	2
Christensen et al, 2019 (29)	Bladder	NGS	Tumor informed	66	NA	NA	NA	NA	surgery	NA	NA	NA	45.8	97.1	3.2

NGS- Next generation sequencing, PCR- polymerase chain reaction, NA- not available

Supplementary Table 4: Surveillance Studies

Study details	Primary site	Testing methodology	Panel type	No of patients	Median Age (Years)	Smoking (%)	Proportion of males (%)	Proportion of >StageIII (%)	Primary treatment	Proportion receiving adjuvant chemo (%)	Median no of surveillance draws	Sensitivity (%)	Specificity (%)	Median lead time (months)
Chaudhuri et al, 2017 (3)	Lung	NGS	Tumor Agnostic	40	66.5	88	68	64	surgery	70	Till relapse	100	100	5.2
Qiu et al, 2021 (37)	Lung	NGS	Tumor informed	89	64	59	65	47	surgery	70		79.4	92.7	NA
Moding et al, 2020 (16)	Lung	NGS	Tumor informed	28	68.5	80	53.8	90.8	surgery	0	NA	100	100	4.1
Waldeck et al, 2022 (25)	Lung	NGS	Tumor informed	21	70	100	67	86.7	surgery	42.8	9	55.5	83.3	2.5
Abbosh et al, 2017 (31)	Lung	NGS	Tumor informed	24		NA			surgery			92.85	90	2.3
Peng et al, 2020 (36)	Lung	NGS	Tumor agnostic	77	60.3	36.4	72.7	20.7	surgery	24.7	5	63.3	61.9	12.6
Chen et al, 2021(5)	Colorectal	NGS	Tumor Informed	240	60	NA	56.7	53.3	surgery	72.5	NA	82.6	94.1	5
Tie et al, 2016 (38)	colorectal	NGS	Tumor informe	178	65	NA	57	0	surgery		8	40.74	98	5.5

			d											
Faikh et al, 2022 (32)	Colorectal	NGS	Tumor informed	48	60	NA	58.3	67	surgery	33	12	53.3	100	0.7
Wang et al, 2019 (39)	Colorectal	NGS	Tumor Informed	58	69	NA	59	48.2	surgery	31	12	100	93.75	3
Henriksen et al, 2022 (10)	Colorectal	NGS	Tumor informed	160	NA	NA	59.3	NA	surgery	86.9	6	88	98.8	10
Parikh et al, 2021 (17)	Colorectal	NGS	Tumor agnostic	84	60	NA	60.7	66.6	surgery	45	Till relapse	70.4	95.8	NA
Reinert et al, 2019 (18)	Colorectal	NGS	Tumor informed	125	70	NA	58.4	64.8	surgery	77	12	87.5	98.3	8.7
Scholer et al, 2017 (20)	Colorectal	PCR	Tumor informed	26	NA	NA	NA	NA	surgery	32.6	8	100	100	9.4
Tarazona et al, 2019 (22)	Colorectal	PCR	Tumor informed	94	71	NA	64.9	41	surgery	43.3	9	77.7	96.5	11.5
Wang et al, 2022 (26)	Colorectal	NGS	Tumor informed	91	59	NA	51.6	NA	surgery	92	12	100	93.75	
Coombes et al, 2019 (6)	Breast	NGS	Tumor Informed	49	57	NA	0	67	surgery	65	8	88.9	100	8.9
Garcia Murillas et	Breast	PCR	Tumor Inform	55	NA	NA	0	NA	surgery	0	5	80	96.4	7.9

al, 2015 (8)			ed											
Garcia Murillas, 2019 (34)	Breast	PCR	Tumor informed	101	54	NA	0			16.8	12	79	91.6	10.7
Olsson et al, 2015 (35)	Breast	PCR	Tumor informed	20	56.5	NA	0	15	surgery	0	6	92.8	100	11
Groot et al, 2019 (9)	Pancreas	PCR	Tumor Informed	59	70	NA	53	27	surgery	41	5	90	88	2.8
Tan et al, 2019 (21)	Melanoma	PCR	Tumor informed	68	57	NA	66	100	surgery	24	6	66.6	92.1	2
Yang et al, 2020 (27)	GE	NGS	Tumor informed	46	54	NA	83	50	surgery	98	NA	84.2	96	6
Christensen et al, 2019 (29)	Bladder	NGS	Tumor informed	66		NA			surgery			100	92.2	3.2
Flach et al, 2022 (33)	Head and Neck	NGS	Tumor informed	17	63	47.1	76.5		surgery			100	100	

NGS- Next generation sequencing, PCR- polymerase chain reaction, NA- not available

Supplementary Table 5: Testing Methodology Details:

	Testing methodology	Panel type	Technology	No of target genes	No of target SNVs/regions/exons	No of SNVs require to define ctDNA+	Depth of assay	LOD of assay
Azad et al, 2020 (1)	NGS	Tumor informed	CAPP-Seq (capture based NGS)	607	802	>=2	10,000X	0.025%
Benhaim et al, 2021 (2)	PCR	Tumor informed	Digital droplet PCR	5 genes (KRAS, BRAF, NRAS, PIK3CA, TP53) and 2 methylation markers (WIF1 and NPY)				
Chaudhuri et al, 2017 (3)	NGS	Tumor Agnostic	CAPP-Seq (capture based NGS)	128		>=5	10,000X	0.025%
Chen et al, 2019 (4)	NGS	Tumor Agnostic	Single molecule amplification and sequencing technology (cSMART)	8	64		20,000X	0.01%
Chen et al, 2021(5)	NGS	Tumor informed	Geneseq Prime (capture based NGS)	425			4693X	
Coombes et al, 2019 (6)	NGS	Tumor Informed	SIGNATERA (amplicon based NGS)		16		100,000 X	0.01%
Diehl et al, 2008 (7)	PCR	Tumor Agnostic	BEAMing and real time PCR	4	27			0.18%
Garcia Murillas et al, 2015 (8)	PCR	Tumor Informed	Digital droplet PCR	14		>2	20,000X	
Groot et al, 2019 (9)	PCR	Tumor Informed	Digital droplet PCR (RAINDANCE PCR system)	1 (KRAS only)	4		20,000 droplets/ variant	0.01%
Henriksen et	NGS	Tumor	SIGNATERA		16	>2	>100,000	0.01%

al, 2022 (10)		informe d	(amplicon based NGS)				X	
Jiang et al, 2020 (11)	NGS	Tumor Agnostic	Capture NGS (Illumina)	1017			1323.8x	
Jin et al, 2020 (12)	PCR	Tumor Agnostic	qPCR (methylation assay)	1 (SEPT9)	10			0.05%
Khakoo et al, 2019(13)	PCR	Tumor Informe d	Digital droplet PCR	6			40,000 droplets/ variant	0.0426%
Lee et al, 2019 (14)	NGS	Tumor informe d	Safe Seq NGS (amplicon based NGS)	1 (KRAS only)				0.1%
McDuff et al, 2021 (15)	NGS	Tumor informe d	Digital droplet PCR					0.01%
Moding et al, 2020 (16)	NGS	Tumor informe d	CAPP-Seq (capture based NGS)			4-8	10,000X	0.025%
Parikh et al, 2021(17)	NGS	Tumor agnostic	Guardant Reveal test (Capture NGS)	73				
Reinert et al, 2019 (18)	NGS	Tumor informe d	SIGNATERA (amplicon based NGS)		16	>2	>100,000 X	0.01%
Sausen et al, 2015(19)	PCR	Tumor informe d	Digital droplet PCR (bioRAD qx 200)	3 (KRAS, BRAP or PIK3CA)			40,000X	0.01%
Scholer et al, 2017 (20)	PCR	Tumor informe d	Digital droplet PCR (bioRAD qx 200)		110		40,000X	0.08%
Tan et al, 2019 (21)	PCR	Tumor informe d	Digital droplet PCR (bioRAD qx 200)	1 (TERT promoter mutation)			40,000X	
Tarazona et al, 2019 (22)	PCR	Tumor informe d	Digital droplet PCR bioRAD qx 100	29 genes			20,000X	
Tie et al, 2019 (24)	NGS	Tumor informe d	Safe-Seq NGS (error education technology)	15		1		
Tie et al, 2019 (23)	NGS	Tumor informe	Safe-Seq NGS (error education	15		1		

		d	technology)					
Waldeck et al, 2022 (25)	NGS	Tumor informed	Capture based NGS	18	52			0.001%
Wang et al, 2021 (26)	NGS	Tumor agnostic	Capture based NGS	451			20,000X	
Yang et al, 2020 (27)	NGS	Tumor informed	Capture NGS (ER-Seq-Enrich rare Mutation Sequencing)	1021		3		
Zviran et al, 2020 (28)	NGS	Tumor informed	Whole genome sequencing (MRDetect)				30X	0.001%
Christensen et al, 2019 (29)	NGS	Tumor informed	SIGNATERA (amplicon based NGS)		16	>=2	>100,000X	0.01%
Abbosh et al, 2017 (31)	NGS	Tumor informed	Multiplex PCR NGS (M-seq)		18	>=2		0.1%
Fakih et al, 2022 (32)	NGS	Tumor informed	SIGNATERA (amplicon based NGS)		16	>=2	>100,000X	0.01%
Flach et al, 2022 (33)	NGS	Tumor informed	Multiplex PCR with target NGS (RaDaR)		21	>=1		0.0011%
Garcia Murillas, 2019 (34)	PCR	Tumor informed	Digital droplet PCR	14		>=2	20,000X	
Olsson et al, 2015 (35)	PCR	Tumor informed	Digital droplet PCR (detection of chromosomal rearrangements)	4-6			Approx. 25,000X	0.01%
Peng et al, 2020 (36)	NGS	Tumor informed	cSMART	127				
Qiu et al, 2021 (37)	NGS	Tumor informed	Ultra-deep sequencing using Capture NGS (Automated Triplet Groom sequencing	139		>=1	30,000X	

			(ATG-Seq)					
Tie et al, 2016 (38)	NGS	Tumor informed	Safe-Seq NGS	15		1		
Wang et al, 2019 (39)	NGS	Tumor Informed	Safe-Seq NGS	15		1		
Parsons et al, 2021 (30)	NGS	Tumor Informed	Duplex sequencing		57	>=2		

NGS- Next generation sequencing, PCR- polymerase chain reaction, NA- not available, LOD-limit of detection

Supplementary Table 6: Subgroup analysis for Sensitivity and Specificity

**Landmark Analysis**				
	Sensitivity rank	P value	Specificity	P value
**Timing of Landmark analysis**				
Pre-chemotherapy	9.07	0.52	8.7	0.23
Post Chemotherapy	11		12.2	
**Panel**				
Tumor informed	14.4	0.21	16.4	0.22
Tumor Agnostic	21.1		11.2	
**Disease sites**				
Lung	24.1	0.12	13	0.23
Breast	9		22.7	
Colorectal	15.5		14	
Pancreas	13.5		12.1	
Others	11.8		21.8	
**Surveillance Analysis**				
	Sensitivity rank	P value	Specificity	P value
**Timing of Surveillance analysis**				
Tumor Informed	12.79	0.62	12.7	0.69
Tumor Agnostic	10.50		11	
**Disease sites**				
Lung	12.83	0.73	9.5	0.33
Breast	12.50		15	
Colorectal	10.78		14.89	
Others	15.20		9.8	

Supplementary Table 7: Meta-regression for colorectal cancer studies (Landmark analysis)

	**Colorectal cancer**		
**Variable**	**Beta coefficient**	***p*-value**	**N studies**
Median Age	-0.42	0.18	12
Proportion of Males	-0.15	0.63	13
Smoking	-	-	-
>=Stage III	0.45	0.17	11
Time to Landmark	0.17	0.58	13
% receiving adjuvant chemotherapy	-0.12	0.36	12
%receiving NACT	-0.98	0.12	3
Baseline ctDNA positivity	0.09	0.75	13

Supplementary Table 8: Meta-regression for sensitivity and specificity (Landmark Analysis)

	**Sensitivity**			**Specificity**		
**Variable**	**Beta coefficient**	***p*-value**	**N studies**	**Beta coefficient**	***p*-value**	**N studies**
Median Age	0.21	0.3	26	-0.28	0.17	26
Proportion of Males	0.24	0.23	28	-0.99	0.33	28
Smoking	-0.58	0.31	5	0.76	0.13	5
>=Stage III	0.26	0.23	23	0.28	0.2	23
Time to Landmark	-0.006	0.98	27	0.28	0.16	27
% receiving adjuvant chemotherapy	0.08	0.73	23	-0.31	0.15	23
%receiving NACT	0.12	0.79	7	-0.573	0.18	6
Baseline ctDNA positivity	0.54	0.005	26	-0.32	0.12	26

NACT-neoadjuvant chemotherapy

Supplementary Table 9: Meta-regression for sensitivity and specificity (Surveillance Analysis)

	**Sensitivity**			**Specificity**		
**Variable**	**Beta coefficient**	***p*-value**	**N studies**	**Beta coefficient**	***p*-value**	**N studies**
Median Age	0.06	0.8	19	0.63	0.54	19

Proportion of Males	-0.25	0.27	21	-0.32	0.15	21
>=Stage III	0.36	0.16	17	0.30	0.24	17
No of surveillance draws	0.24	0.38	15	0.55	0.03	15
% receiving adjuvant chemotherapy	0.39	0.17	18	0.49	0.038	18
%receiving NACT	0.076	0.89	6	-0.15	0.78	6
Baseline ctDNA positivity	0.32	0.16	21	0.35	0.12	21

NACT-neoadjuvant chemotherapy

Supplementary Figure 1: Landmark Analysis by timing of sample with respect to adjuvant chemotherapy

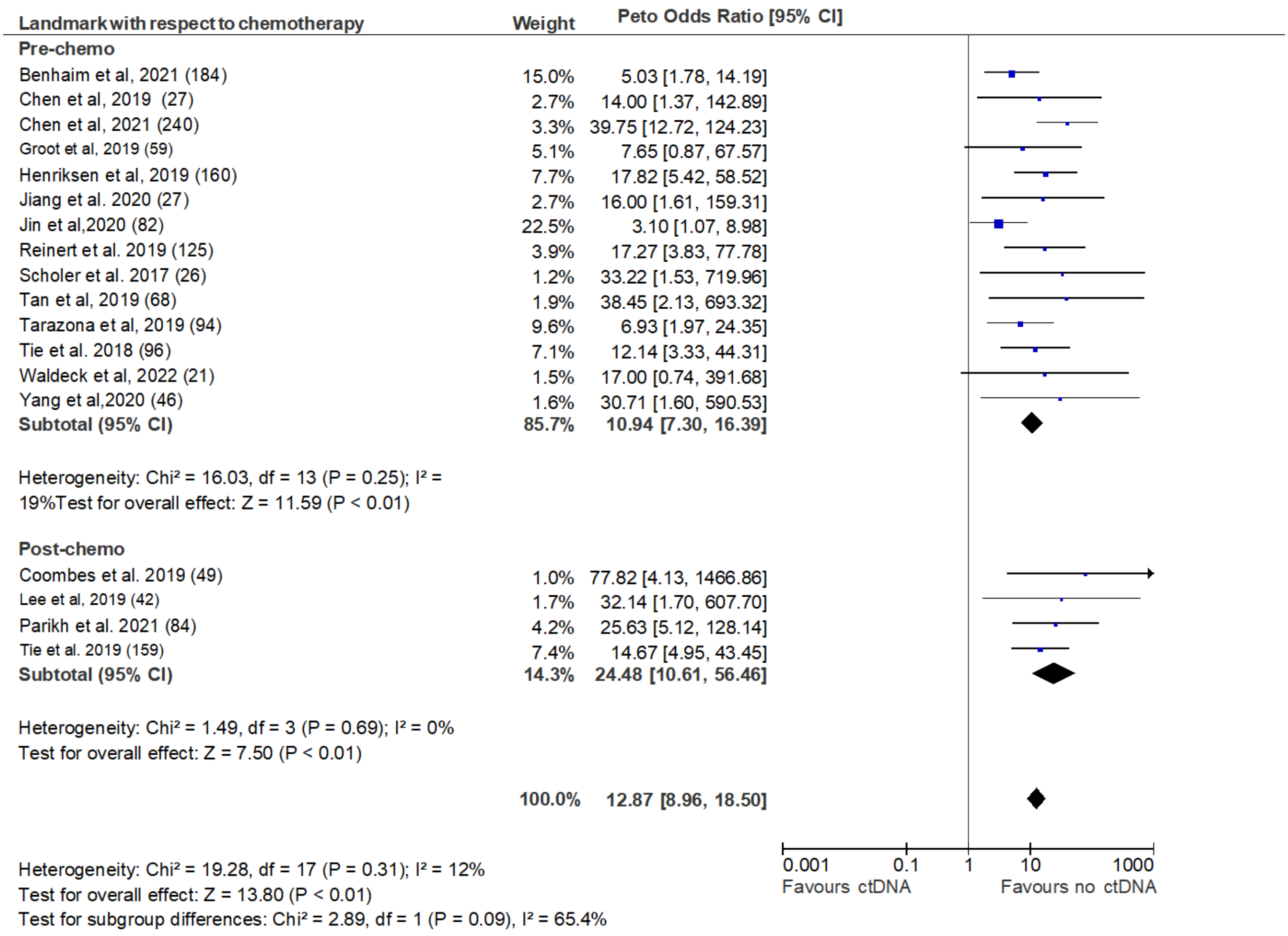

(N)= no of patients

Supplementary Figure 2: Funnel plot for landmark studies

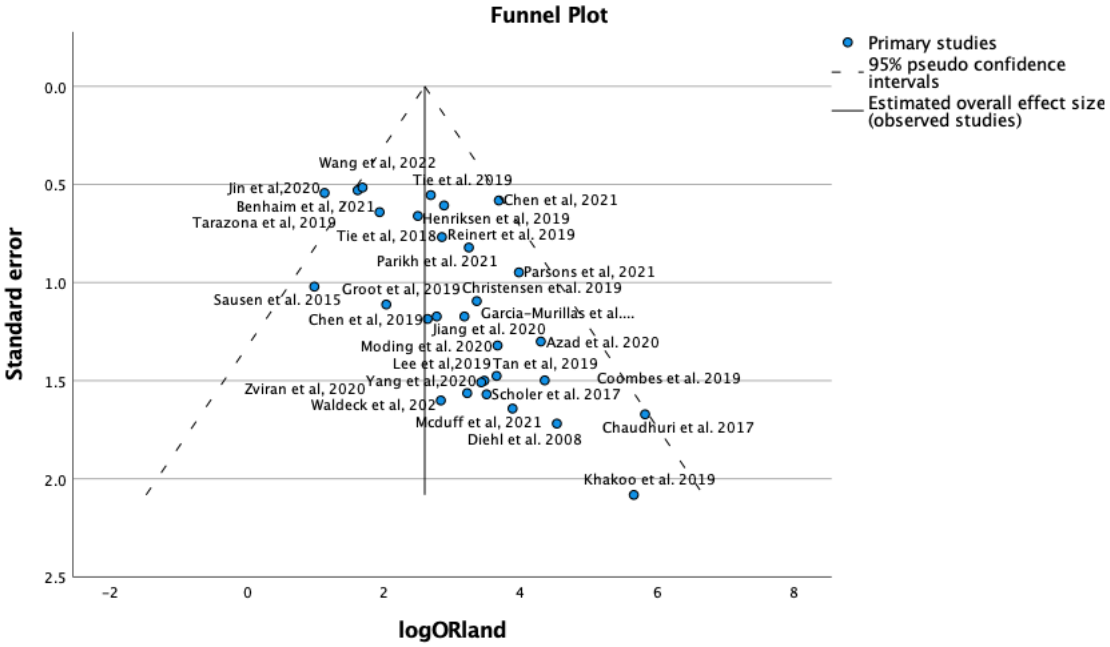

Supplementary Figure 3: Surveillance by disease site (sensitivity analysis)

Surveillance-type of panel	Weight	Peto Odds Ratio [95% CI]
**Tumor informed**		
Abbosh et al. 2017 (24)	2.7%	26.23 [5.24, 131.41]
Chen et al, 2021 (240)	5.5%	116.40 [37.73, 359.08]
Christensen et al. 2019 (66)	3.8%	104.64 [26.64, 410.95]
Coombes et al. 2019 (49)	3.5%	28.51 [6.85, 118.72]
Faikh et al, 2022 (48)	2.7%	12.43 [2.47, 62.45]
Flach et al, 2022 (63)	1.4%	93.07 [10.10, 858.02]
Garcia-Murillas et al. 2015 (55)	2.3%	18.50 [3.27, 104.71]
Garcia-Murillas et al. 2019 (101)	7.8%	30.98 [12.00, 79.95]
Groot et al, 2019 (59)	4.6%	25.90 [7.47, 89.78]
Henriksen et al, 2019 (160)	5.5%	244.28 [78.44, 760.73]
Moding et al. 2020 (28)	2.2%	112.73 [18.97, 669.98]
Olsson et al. 2015 (20)	1.8%	48.31 [6.84, 340.99]
Qiu et al, 2021 (89)	8.8%	23.16 [9.49, 56.53]
Reinert et al. 2019 (125)	2.5%	48.42 [8.89, 263.54]
Scholer et al. 2017 (26)	3.1%	47.90 [10.51, 218.26]
Tan et al, 2019 (68)	7.5%	12.68 [4.81, 33.43]
Tarazona et al, 2019 (94)	4.2%	82.54 [22.75, 299.43]
Tie et al. 2016 (178)	3.1%	203.95 [44.74, 929.75]
Waldeck et al, 2022 (21)	2.2%	5.29 [0.88, 31.69]
Wang et al. 2019 (58)	2.7%	199.95 [39.58, 1010.00]
Yang et al,2020 (46)	4.8%	27.28 [8.13, 91.60]
**Subtotal (95% CI)**	**82.6%**	**42.84 [32.00, 57.36]**
Heterogeneity: Chi² = 42.44, df = 20 (P = 0.002); I² = 53%, Test for overall effect: Z = 25.24 (P < 0.01)		
**Tumor agnostic panel**		
Chaudhuri et al. 2017 (40)	3.7%	39.41 [9.94, 156.28]
Parikh et al. 2021 (84)	5.7%	15.32 [5.03, 46.67]
Peng et al. 2020 (77)	8.0%	1.84 [0.72, 4.69]
**Subtotal (95% CI)**	**17.4%**	**7.04 [3.73, 13.31]**
Heterogeneity: Chi² = 15.78, df = 2 (P = 0.004); I² = 87% Test for overall effect: Z = 6.02 (P < 0.01)		
	**100.0%**	**31.30 [24.01, 40.80]**
Heterogeneity: Chi² = 83.80, df = 23 (P < 0.01); I² = 73% Test for overall effect: Z = 25.45 (P < 0.01) Test for subgroup differences: Chi² = 25.57, df = 1 (P < 0.01), I² = 96.1%		

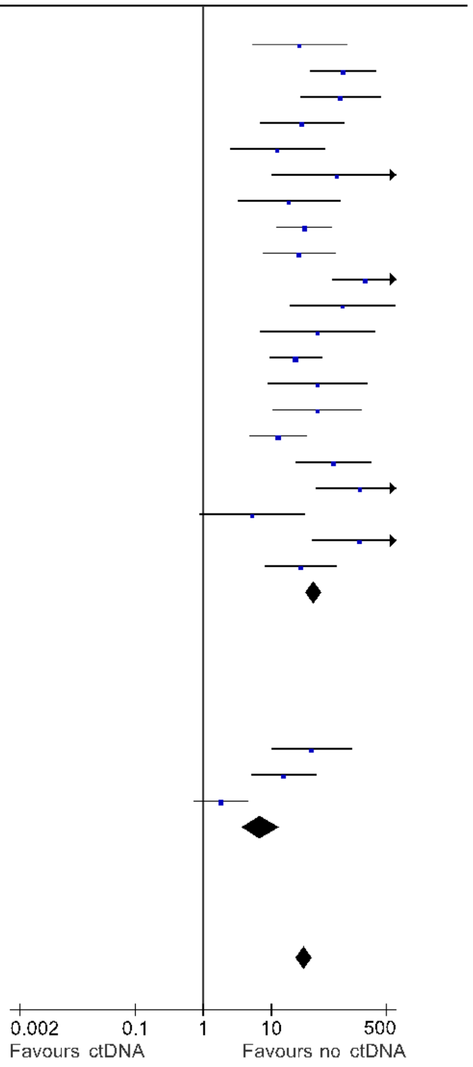

(N)= number of patients

Supplementary Figure 4: Funnel Plot for surveillance studies

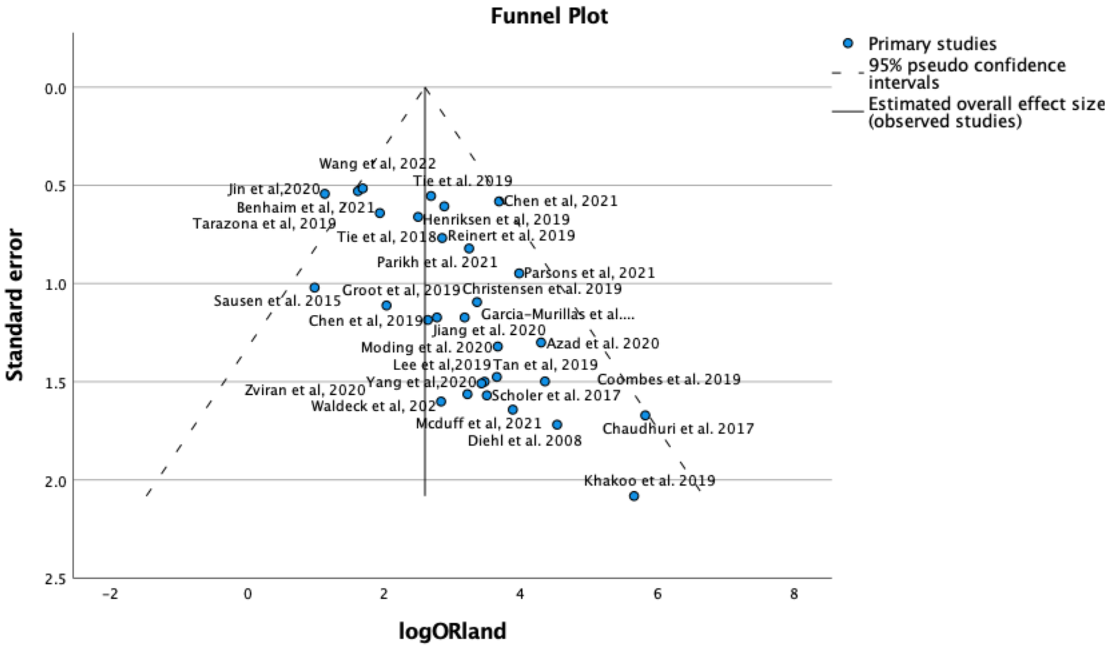

Supplement: pkad040_Supplementary_Data [file pkad040_supplementary_data.pdf]
